# Bilateral Choroidal Neovascular Membranes in a Patient With a C1QTNF5 Mutation Managed With Long‐Term Anti‐VEGF Therapy and PRN Protocol: A Case Report

**DOI:** 10.1155/crop/6047720

**Published:** 2026-09-15

**Authors:** Zishuai Chou, Celine Saade

**Affiliations:** ^1^ Division of Ophthalmology, The Warren Alpert Medical School of Brown University, Providence, Rhode Island, USA, brown.edu; ^2^ Brown University Health, Providence, Rhode Island, USA

**Keywords:** anti-VEGF therapy, C1QTNF5 mutation, choroidal neovascular membrane, PRN protocol

## Abstract

Choroidal neovascular membranes (CNVM) are abnormal vascular complexes that can cause serious visual morbidity if untreated. They can be induced by the C1QTNF5 mutation, which disrupts retinal pigment epithelium (RPE) adhesion and leads to progressive sub‐RPE deposit accumulation. Due to its overlapping clinical presentation with age‐related macular degeneration (AMD), C1QTNF5‐associated CNVM is often misdiagnosed. Early recognition of atypical CNVM clinical presentations is essential to prompt genetic testing and initiate personalized antivascular endothelial growth factor (anti‐VEGF) therapy. We report a 57‐year‐old male with a history of pontine stroke and strabismus surgery who presented with bilateral submacular hemorrhages and visual distortion. Given the atypical bilateral presentation and absence of drusen, genetic testing was performed. A pathogenic C1QTNF5 mutation with a p. Asn22Lys variant was identified. Treatment was initiated with intravitreal bevacizumab (IVA) injection and switched to aflibercept (IVE). After 2 years of tailored injection intervals based on optical coherence tomography (OCT) and visual acuity, the patient achieved visual acuity of 20/25 in both eyes with complete or near‐complete resolution of subretinal fluid. A pro re nata (PRN) protocol was then initiated, and vision remained stable at the 4‐year follow‐up. This case highlights the importance of considering inherited retinal disease in atypical CNVM presentations. Early diagnosis, genetic testing, and individualized, imaging‐driven anti‐VEGF therapy can lead to long‐term stability and favorable visual outcomes in patients with C1QTNF5‐associated CNVM. Although causality cannot be established from a single case, concurrent glaucoma findings support the need for comprehensive ocular surveillance in patients with C1QTNF5 mutations.

## 1. Introduction

Choroidal neovascular membranes (CNVM) are retinal disruptions resulting from abnormal vascular complexes forming in the choroid [1]. If untreated, they can cause subretinal scarring and damage photoreceptors, leading to irreversible vision loss [2]. CNVM are commonly associated with age‐related macular degeneration (AMD), myopic degeneration, inflammation, or ocular trauma [3–5]. The risk of CNVM increases with age, with the majority of cases in individuals over the age of 60 [3]. Thus, genetic etiologies need to be considered when CNVM occurs in younger patients, in patients without typical risk factors, or those with atypical presentations [3, 4].

One of the genetic causes, *C1QTNF5* gene mutation, is often associated with an autosomal dominant disorder called late‐onset retinal degeneration (L‐ORD) [6]. This mutation disrupts retinal pigment epithelium (RPE) adhesion and creates progressive sub‐RPE deposits, leading to CNVM formation and photoreceptor degeneration [6, 7]. Multiple pathogenic C1QTNF variants have been described, including p. Ser163Arg, p. Pro188Thr, and p. Pro188Leu, each with different clinical presentations [6–10]. Common presentations of C1QTNF5 mutation are nyctalopia, gradual central vision loss, and retinal atrophy, but may also have anterior segment changes, including long anterior zonules (LAZ) and iris atrophy. Because of its overlapping features with those of AMD, C1QTNF5 mutation′s early‐stage presentation can often be misdiagnosed [7]. Hence, early recognition of atypical CNVM presentations and genetic testing are critical for accurate diagnosis [8]. Although antivascular endothelial growth factor (anti‐VEGF) therapy is the main CNVM treatment, its therapeutic course for C1QTNF5‐associated CNVM can differ in duration and frequency [9].

Here, we describe a rare case of bilateral CNVM associated with a pathogenic p. Asn22Lys *C1QTNF5* mutation in a 57‐year‐old male over the course of 4 years. To our knowledge, a review of the available literature did not identify any previously reported cases of CNVM associated with this specific variant. Its clinical course, diagnostic workup, and management demonstrate the significance of early identification, genetic testing, personalized anti‐VEGF therapy, and long‐term monitoring in patients with C1QTNF5‐associated disease. Its potential association with glaucoma and future therapeutics with gene editing are also discussed.

## 2. Case Presentation

A 57‐year‐old male with a history notable for pontine stroke 1 year before presentation, with diplopia that was treated with bilateral strabismus surgery, presented with a 2‐week onset of constant right eye vision distortion, described by the patient as “dullness in the central visual area.” Social history was significant for former tobacco use and ongoing medical marijuana use, and family history was unremarkable for ocular conditions.

At initial visit, his distant visual acuity (VA) was 20/80 in the right eye (OD) and 20/20 in the left eye (OS), with near VA 20/400 OD and 20/50 OS. Intraocular pressure (IOP) was 20‐mmHg OD and 21‐mmHg OS. Amsler grid eye test found wavy lines seen in OD.

**Figure 1:**
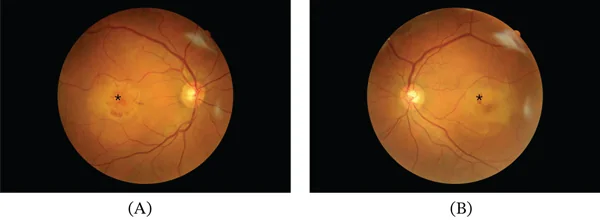
Baseline color fundus photo of both eyes (OU) from the first visit on December 30, 2020. (A) OD and (B) OS. Bilateral submacular hemorrhages (∗) in the absence of visible drusen.

**Figure 2:**
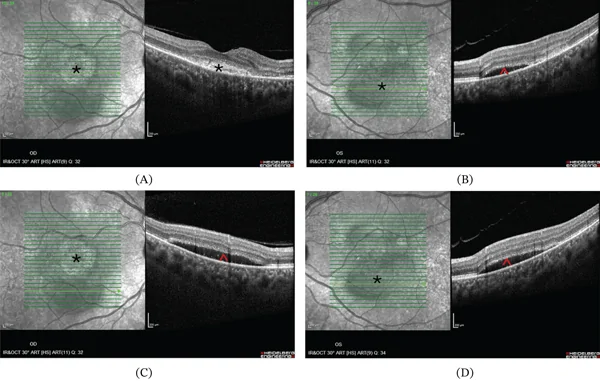
Baseline OCT OU from the first visit on December 30, 2020. (A and B) OD and (C and D) OS. Active CNVM (∗) with overlying subretinal fluid (**^**) OU.

**Figure 3:**
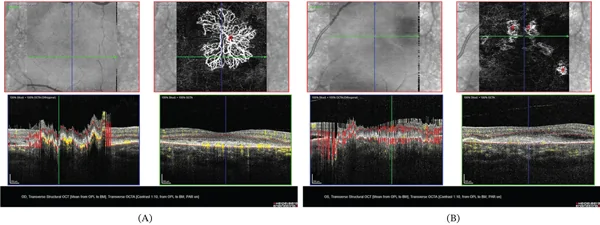
Baseline transverse OCTA OU from the first visit on December 30, 2020. (A) OD and (B) OS. Abnormal vascular complex (**#**) between Bruch′s membrane and RPE OU.

Dilated slit‐lamp examination was notable for macular subretinal hemorrhages without drusen in both eyes (OU). Fundus photography and fluorescein angiography (FA) showed subfoveal hemorrhage (Figure 1). Optical coherence tomography (OCT) and optical coherence tomography with angiography (OCTA) confirmed the presence of CNVM with SRF and active choroidal vascular complexes (Figures 2 and 3). Differential diagnoses included bilateral CNVM (idiopathic or genetic), Sorsby macular dystrophy, and less likely AMD or polypoidal choroidal vasculopathy (PCV).

Treatment was initiated with intravitreal bevacizumab injection (IVA) OU every 4–5 weeks, with improvement of bilateral macular hemorrhage and VA. Five months later, given a partial response to IVA (persistent SRF and ongoing CNVM activity on OCT), treatment was switched to intravitreal aflibercept injection (IVE) to aim for a complete response. Given the atypical presentation of CNVM with an early onset, lack of drusen or trauma, and bilateral nature, genetic etiology was suspected. Genetic testing was ordered for genetic macular and retinal dystrophies and found a C1QTNF5 mutation with a p. Asn22Lys variant, which affects RPE cell adhesion and polarity. Family variant testing was offered, and the patient′s sister was found to have the same mutation.

**Figure 4:**
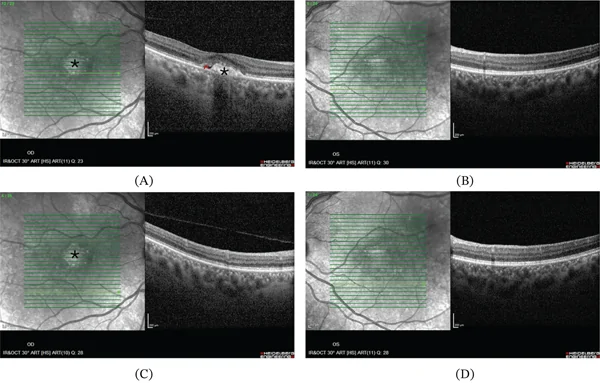
Follow‐up OCT after long‐term anti‐VEGF therapy from the latest visit on September 22, 2025. (A) OD and (B) OS. (A and C) Flattened CNVM (∗) with chronic trace SRF (^) OD. (B and D) Flattened CNVM and resolution of SRF OS.

Over 4 years, the patient received 14 injections based on disease activity on clinical examination, OCT, and OCTA (Table 1). Given SRF resolution, flattening of CNVM with residual scarring, and VA improvement, treatment intervals were gradually extended. Given no new SRF and stable CNVM upon extending the treatment to a 3‐month interval, the patient was switched to a PRN protocol. Retreatment during the PRN phase was guided by OCT evidence of new or worsening SRF, enlargement of CNVM, new hemorrhage, or decline in VA. SRF had largely resolved, and distant VA had improved to approximately 20/25 OU (Table 2), with the most recent follow‐up in September 2025 (Figure 4).

**Table 1:** Longitudinal clinical course and treatment timeline.

Phase	Time period	Key clinical findings	Management
Initial presentation and induction therapy	December 2020–May 2021	Bilateral CNVM with submacular hemorrhage and SRF without drusen. Persistent CNVM activity despite initial improvement with IVA.	Initiated IVA OU every 4–5 weeks, later transitioned to IVE OU for persistent SRF and ongoing CNVM activity.
Genetic evaluation and interval extension	July 2021–June 2022	Genetic testing identified pathogenic C1QTNF5 p. Asn22Lys variant. Progressive reduction in SRF and hemorrhage with fibrotic CNVM stabilization.	IVE intervals progressively extended based on OCT findings and clinical response.
Initial PRN phase	October 2022–August 2023	Stable trace SRF with improving CNVM and stable visual acuity.	Transitioned to PRN monitoring guided by OCT findings and visual acuity changes.
Recurrence and retreatment	September 2023–July 2024	Recurrent SRF and enlarged CNVM OS with intermittent hemorrhage.	Resumed IVE OS with subsequent anatomical improvement and SRF resolution.
Long‐term stabilization	August 2024–September 2025	Stable CNVM anatomy with no or trace SRF and maintained visual acuity approximately 20/20–20/25 OU.	Continued PRN surveillance with periodic OCT monitoring and individualized retreatment.

**Table 2:** Longitudinal visual acuity and intraocular pressure measurements.

Date	VA OD	VA OS	IOP OD	IOP OS
December 30, 2020	20/80	20/20	20	21
January 08, 2021	20/60 −1	20/25 −2	20	23
February 05, 2021	20/60 −1	20/40 −2	20	22
March 05, 2021	20/60 −1	20/30 +3	19	21
April 23, 2021	20/70 −2	20/40 −2	18	21
May 20, 2021	20/200	20/100	28	23
November 04, 2021	20/150	20/100	21	24
December 30, 2021	20/100	20/100	15	17
February 17, 2022	20/150	20/70	15	15
June 28, 2022	20/20	20/20 −2	Not recorded	Not recorded
October 05, 2022	20/20	20/30	17	18
November 04, 2022	20/30	20/30	16	16
January 13, 2023	20/40	20/25 +2	15	15
March 15, 2023	20/40	20/30 −1	23	24
May 18, 2023	20/30	20/30 −1	10	10
September 22, 2023	20/20	20/20	14	15
November 01, 2023	20/30	20/20 −2	15	13
December 15, 2023	20/20	20/20	Not recorded	Not recorded
January 16, 2024	20/25 +1	20/20 −1	10	11
February 21, 2024	20/20	20/20 −2	12	13
March 27, 2024	20/30 −2	20/25 −1	15	14
May 22, 2024	20/25 −1	20/25 −1	13	16
July 08, 2024	20/20 −1	20/20	14	15
August 14, 2024	20/25 −1	20/40	15	14
October 14, 2024	20/25	20/25	15	16
January 14, 2025	20/25	20/25 −2	14	15
June 4, 2025	20/40	20/20	18	16
July 9, 2025	20/20 −3	20/20	14	16
September 22, 2025	20/20	20/20 −2	10	12

While undergoing the longitudinal retinal treatment, the patient was suspected of glaucoma OU due to a mild cup/disc ratio elevation (OS > OD) with IOP toggling near the upper limits of normal (21–24 mmHg) over multiple visits (Table 2). Glaucoma evaluation found mild primary open‐angle glaucoma OU with normal corneal thickness. Latanoprost was initiated, and he has been managed by a glaucoma specialist with no evidence of disease progression to date.

## 3. Discussion

In our patient with bilateral C1QTNF5‐associated CNVM, personalized anti‐VEGF therapy has yielded bilateral anatomical and functional stability over 4 years, with the most recent best corrected visual acuity (BCVA) of 20/20–20/25 OU. A PRN protocol enabled individualized retreatment based on OCT findings. This approach demonstrates the potential efficacy of a personalized, exam and imaging‐driven management of genetically associated CNVM.

Multiple published reports have described CNVM in *C1QTNF5* mutation carriers with variable clinical courses [7–12] (Table 3). Vincent et al. [9] reported sequential bilateral CNVM in a family with the p. Ser163Arg variant. Torrell‐Belzach et al. [7] described a patient with the p. Pro188Leu variant undergoing anti‐VEGF therapy. To our knowledge, a review of the available literature did not identify any previously reported cases of CNVM associated with the p. Asn22Lys C1QTNF5 variant. The present case therefore expands the phenotypic spectrum of C1QTNF5‐associated disease and demonstrates successful long‐term management with individualized anti‐VEGF therapy and a PRN protocol. Additionally, the concurrent glaucoma findings observed during follow‐up further support the need for comprehensive ocular surveillance in these patients. The combination of bilateral CNVMs, early onset, and a lack of drusen prompted genetic testing. Those atypical presentations should raise suspicion for inherited retinal dystrophies.

**Table 3:** Case reports/series of C1QTNF5‐related CNVM.

Study	Type of study	Variant	Number of patients with CNVM	Age of CNVM onset	CNVM presentation	Therapy	Note
Soumplis et al. [12]	Case series	Ser163Arg	2	66 years, 64 years		None reported	Four unrelated Caucasian patients.
Aye et al. [11]	Case report	Ser163Arg	1	61 years	Unilateral CNVM	Anti‐VEGF	Treated with intravitreal ranibizumab.
Vincent et al. [9]	Case series	Ser163Arg	2	63 years, 68 years	Bilateral CNVM in two family members	None reported	Patients from the same family. Regression of CNVM noted in one patient.
De Zaeytijd et al. [8]	Case series	Pro188Thr	2	60 years, 64 years	Patient 1: unilateral CNVM, Patient 2: not specified	Patient 1: anti‐VEGF, Patient 2: not treated	Patient 2 had CNVM before VEGF therapy was available.
Torrell‐Belzach et al. [7]	Case report	Pro188Leu	1	66 years	Bilateral CNVM, OS > OD	Anti‐VEGF	11‐year follow‐up.

It is notable that our patient developed signs of suspected glaucoma during follow‐up. Although glaucoma is not traditionally related to genetically associated CNVM, emerging evidence suggests a possible association between C1QTNF5‐associated disease and glaucoma‐related findings due to their predisposition to anterior segment changes. A prospective cohort study by Lando et al. [13] reported consistent anterior segment abnormalities in a patient with a p. Ser163Arg *C1QTNF5* mutation, where LAZ and iris atrophy occurred before any fundus manifestation, and they coexisted with open anterior chamber angles and variable trabecular pigmentation Similarly, Vincent et al. [9] described that LAZ may elevate the risk of IOP elevation in individuals with a *C1QTNF5* mutation. De Zaeytijd et al. [8] also observed iris abnormalities and poor dilation in patients with a p. Pro188Thr *C1QTNF5* mutation, although the glaucoma incidence was low. Although causality cannot be established, these findings raise the possibility that the C1QTNF5 mutation may impact anterior chamber anatomy and aqueous outflow, potentially contributing to susceptibility to glaucoma. In our patient, glaucoma risk was noticed via IOP readings near the upper limits of normal over multiple visits, accompanied by an increase in cup/disc ratio. Prompt evaluation and latanoprost treatment controlled disease progression. This case suggests the importance of comprehensive longitudinal ocular monitoring, including optic nerve evaluation and IOP surveillance, in patients with C1QTNF5‐associated disease. Overall, there is a substantial need for more clinical and basic science investigations on the link between the C1QTNF5 mutation and glaucoma, as well as clinical management guidelines accounting for glaucoma surveillance.

Looking ahead, gene‐editing therapies may be transformative in *C1QTNF5*‐associated CNVM. For instance, a preclinical study by Li et al. [14] illustrated that CRISPR‐based gene correction in a knock‐in mouse model with p. Ser163Arg C1QTNF5 mutation reversed early RPE pathology, reduced sub‐RPE deposits, and preserved photoreceptor function. It was more effective in early disease stages. Although the findings are preliminary without clinical validation, they highlight the future potential of disease‐modifying therapies for C1QTNF5‐associated retinal degeneration, further emphasizing the importance of early disease identification.

Lastly, because of the autosomal dominant inheritance pattern, it is crucial to offer genetic counseling and family screening [14]. Given the variable onset and subclinical retinal changes in early stages, first‐degree relatives of affected individuals should be offered genetic testing and baseline retinal imaging [9, 14]. This ensures early identification, enabling advanced monitoring, timely treatment of CNVM, and potential enrollment in gene therapy trials [6, 10]. Genetic counseling may also help families make informed decisions on reproductive options and long‐term vision planning.

This case highlights the importance of considering inherited retinal dystrophies in patients presenting with atypical clinical features of CNVM. Early recognition, genetic testing, and personalized anti‐VEGF therapy may support favorable long‐term anatomical and functional outcomes in C1QTNF5‐associated CNVM. Although further investigation is needed to clarify any relationship between C1QTNF5‐associated disease and glaucoma, this case supports the value of comprehensive longitudinal ocular surveillance in affected patients, including anterior segment evaluation and IOP monitoring. Gene editing, when implemented early, may be promising for disease modification. This case illustrates how a forward‐thinking, multidisciplinary approach and innovation can improve long‐term vision outcomes in this rare *C1QTNF5*‐associated CNVM.

## Author Contributions

C.S.: study design, data collection of the patient, analysis and interpretation of patient data, manuscript revision, and study supervision. Z.C.: study design, analysis and interpretation of patient data, and drafting the manuscript.

## Funding

No funding was received for this manuscript.

## Consent

Informed consent for publication was obtained from the patient included in the case report.

## Conflicts of Interest

The authors declare no conflicts of interest.

## Data Availability

The data that support the findings of this study are available on request from the corresponding author. The data are not publicly available due to privacy or ethical restrictions.

## References

[1] Chirco K. R. , Sohn E. H. , Stone E. M. , Tucker B. A. , and Mullins R. F. , Structural and Molecular Changes in the Aging Choroid: Implications for Age-Related Macular Degeneration, Eye. (2017) 31, no. 1, 10–25, 10.1038/eye.2016.216, 27716746. 27716746 PMC5233940

[2] Klein M. L. , Jorizzo P. A. , and Watzke R. C. , Growth Features of Choroidal Neovascular Membranes in Age-Related Macular Degeneration, Ophthalmology. (1989) 96, no. 9, 1416–1421, 10.1016/S0161-6420(89)32741-2. 2476700

[3] Pugazhendhi A. , Hubbell M. , Jairam P. , and Ambati B. , Neovascular Macular Degeneration: A Review of Etiology, Risk Factors, and Recent Advances in Research and Therapy, International Journal of Molecular Sciences. (2021) 22, no. 3, 10.3390/ijms22031170, 33504013. PMC7866170 33504013

[4] Spaide R. F. , Choroidal Neovascularization in Younger Patients, Current Opinion in Ophthalmology. (1999) 10, no. 3, 177–181, 10.1097/00055735-199906000-00005. 10537776

[5] Sheth J. U. , Stewart M. W. , and Narayanan R. , Macular Neovascularization, Survey of Ophthalmology. (2024) 70, no. 4, 653–675, 10.1016/j.survophthal.2024.101349. 39222802

[6] Stanton C. M. , Borooah S. , Drake C. , Marsh J. A. , Campbell S. , Lennon A. , Soares D. C. , Vallabh N. A. , Sahni J. , Cideciyan A. V. , Dhillon B. , Vitart V. , Jacobson S. G. , Wright A. F. , and Hayward C. , Novel Pathogenic Mutations in C1QTNF5 Support a Dominant Negative Disease Mechanism in Late-Onset Retinal Degeneration, Scientific Reports. (2017) 7, no. 1, 12147, 10.1038/s41598-017-11898-3, 28939808. 28939808 PMC5610255

[7] Torrell-Belzach N. , Miere A. , Bhouri R. , Srour M. , Souied E. H. , and Zambrowski O. , An Incipient Late-Onset Retinal Degeneration With a C1QTNF5 Mutation: A Case Report With an 11-Year Follow-Up, Documenta Ophthalmologica. (2024) 148, no. 1, 57–64, 10.1007/s10633-023-09958-3, 38129706. 38129706

[8] De Zaeytijd J. , Coppieters F. , De Bruyne M. , and Leroy B. P. , Longitudinal Phenotypic Study of Late-Onset Retinal Degeneration due to a Founder Variant c.562C>A p.(Pro188Thr) in theC1QTNF5gene, Ophthalmic Genetics. (2021) 42, no. 5, 521–532, 10.1080/13816810.2021.1923041. 33949280

[9] Vincent A. , Munier F. L. , Vandenhoven C. C. , Wright T. , Westall C. A. , and Héon E. , The Characterization of Retinal Phenotype in a Family With C1QTNF5-Related Late-Onset Retinal Degeneration, Retina. (2012) 32, no. 8, 1643–1651, 10.1097/IAE.0b013e318240a574, 22277927. 22277927

[10] Borooah S. , Collins C. , Wright A. , and Dhillon B. , Republished review. [corrected] Late-Onset Retinal Macular Degeneration: Clinical Insights Into an Inherited Retinal Degeneration, British Journal of Ophthalmology. (2009) 85, no. 1007, 495–500, 10.1136/bjo.2008.150151, 19734518. 19734518

[11] Aye K. , Gupta R. , Talks S. , and Browning A. C. , Treatment of a Choroidal Neovascular Membrane in a Patient With Late-Onset Retinal Degeneration (L-ORD) With Intravitreal Ranibizumab, Eye. (2010) 24, no. 9, 1528–1530, 10.1038/eye.2010.71, 20489737. 20489737

[12] Soumplis V. , Sergouniotis P. I. , Robson A. G. , Michaelides M. , Moore A. T. , Holder G. E. , and Webster A. R. , Phenotypic Findings in C1QTNF5 Retinopathy (Late-Onset Retinal Degeneration), Acta Ophthalmologica. (2013) 91, no. 3, e191–e195, 10.1111/aos.12010, 23289492. 23289492

[13] Lando L. , Nguyen A. X. L. , Li R. T. H. , Megaw R. , Dhillon B. , and Borooah S. , Anterior Segment Phenotypic Changes in Late-Onset Retinal Degeneration With Ser163Arg Mutation in CTRP5/C1QTNF5, Graefe′s Archive for Clinical and Experimental Ophthalmology. (2023) 261, no. 9, 2507–2516, 10.1007/s00417-023-06041-0, 37043002.37043002

[14] Li R. T. H. , Roman A. J. , Sumaroka A. , Stanton C. M. , Swider M. , Garafalo A. V. , Heon E. , Vincent A. , Wright A. F. , Megaw R. , Aleman T. S. , Browning A. C. , Dhillon B. , and Cideciyan A. V. , Treatment Strategy With Gene Editing for Late-Onset Retinal Degeneration Caused by a Founder Variant in C1QTNF5, Ophthalmology & Visual Science. (2023) 64, no. 15, 10.1167/iovs.64.15.33, 38133503. PMC10746929 38133503

